# Production of hydrophobic amino acids from biobased resources: wheat gluten and rubber seed proteins

**DOI:** 10.1007/s00253-016-7441-8

**Published:** 2016-04-27

**Authors:** Widyarani, Yessie W. Sari, Enny Ratnaningsih, Johan P. M. Sanders, Marieke E. Bruins

**Affiliations:** 1 Biobased Chemistry and Technology, Wageningen University, Bornse Weilanden 9, 6708 WG Wageningen, the Netherlands; 2 Research Centre for Chemistry, Indonesian Institute of Sciences (LIPI), Building 452, Kawasan Puspiptek Serpong, Tangerang Selatan, 15314 Indonesia; 3 Biophysics Division, Department of Physics, Bogor Agricultural University, Kampus IPB Darmaga, Bogor, 16680 Indonesia; 4 Study Programme of Chemistry, Faculty of Mathematics and Natural Sciences, Institut Teknologi Bandung, Jl. Ganesha 10, Bandung, 40132 Indonesia; 5 Food & Biobased Research, Wageningen UR, Bornse Weilanden 9, 6708 WG Wageningen, the Netherlands

**Keywords:** Biorefinery, Protein hydrolysis, Protease, Rubber seed, Wheat gluten, Hydrophobicity

## Abstract

Protein hydrolysis enables production of peptides and free amino acids that are suitable for usage in food and feed or can be used as precursors for bulk chemicals. Several essential amino acids for food and feed have hydrophobic side chains; this property may also be exploited for subsequent separation. Here, we present methods for selective production of hydrophobic amino acids from proteins. Selectivity can be achieved by selection of starting material, selection of hydrolysis conditions, and separation of achieved hydrolysate. Several protease combinations were applied for hydrolysis of rubber seed protein concentrate, wheat gluten, and bovine serum albumin (BSA). High degree of hydrolysis (>50 %) could be achieved. Hydrophobic selectivity was influenced by the combination of proteases and by the extent of hydrolysis. Combination of Pronase and Peptidase R showed the highest selectivity towards hydrophobic amino acids, roughly doubling the content of hydrophobic amino acids in the products compared to the original substrates. Hydrophobic selectivity of 0.6 mol-hydrophobic/mol-total free amino acids was observed after 6 h hydrolysis of wheat gluten and 24 h hydrolysis of rubber seed proteins and BSA. The results of experiments with rubber seed proteins and wheat gluten suggest that this process can be applied to agro-industrial residues.

**Electronic supplementary material:**

The online version of this article (doi:10.1007/s00253-016-7441-8) contains supplementary material, which is available to authorized users.

## Introduction

As the building blocks of proteins, amino acids are important components in food and feed. Alternatively, amino acids can be used for chemicals production to reduce fossil fuel consumption (Scott et al. 2007). From the 20 proteinogenic amino acids, isoleucine, leucine, valine, phenylalanine, tryptophan, methionine, threonine, histidine, and lysine are essential amino acids as they cannot be synthesised by humans and most farm animals. This makes them important in the human and animal diet. From these amino acids, the first six have hydrophobic side chains (Black and Mould 1991). Amino acid hydrophobicity is often defined by its partitioning between two liquid phases (Biswas et al. 2003), and this property can be important in downstream processing. Producing mixtures rich in hydrophobic amino acids is therefore an interesting process to investigate based on the ease in further processing and their potential application as a group in food and feed. This approach increases the feasibility of a biorefinery route from protein to food/feed and bulk chemicals (Sari et al. 2015).

The hydrophobicity of amino acids has been extensively studied as hydrophobic interactions play a dominant role in stabilising protein structures (Tanford 1962; Biswas et al. 2003). Amino acids with hydrophobic side chains tend to reside in the interior of a protein to minimise contact with water. This tendency can be approximated by determining amino acid partition between water and organic phase (Nozaki and Tanford 1971). The partitioning can also be calculated from amino acid solubility in an organic solvent and expressed as free energy changes of transfer from organic solvent to water. With this approach, tryptophan shows to be the most hydrophobic (Tanford 1962; Nozaki and Tanford 1971). Alternatively, the partitioning can be calculated based on phase-partitioning behaviour of molecular fragments that build the amino acid. Phenylalanine is shown as the most hydrophobic amino acid based on this approach (Black and Mould 1991). Despite methods differences, there is a good agreement that the following amino acids: phenylalanine, leucine, isoleucine, tyrosine, tryptophan, valine, methionine, and proline can be grouped as hydrophobic.

Amino acids can be produced by chemical synthesis, fermentation, or protein hydrolysis (Ivanov et al. 2013). Protein hydrolysis has a high potential because the proteins can be obtained from several sources including agro-industrial residues, which include residues from first generation bioethanol or biodiesel production, leaves, grass, stover, microalgae, and animal slaughter waste, with varying protein content from 5 to 90 % (Lammens et al. 2012). Dried distillers grains with solubles (DDGS) is an important by-product from bioethanol production. The weight of DDGS is roughly the same as the produced ethanol (Villegas-Torres et al. 2015). Wheat DDGS contains 36–38 % protein that is predominated by gluten (80–85 % of wheat protein) and has remarkably high (34 %) content of glutamic acid/glutamine (Lammens et al. 2012; Villegas-Torres et al. 2015). The other potential agro-industrial residues are rubber seeds. They are available from rubber tree (*Hevea brasiliensis*) plantations, of which the latex is the main product that is used in natural rubber production. Recently, there are growing interests in using rubber seeds for oil and protein production (Zhu et al. 2014; Widyarani et al. 2014). Rubber seed press cake, the residue after oil pressing, contains 22 % protein that consists of one-third hydrophobic amino acids (Widyarani et al. 2014). With the increasing production of biofuel, the availability of wheat DDGS, rubber seed press cake, and similar residues are expected to increase in the coming years.

Complete protein hydrolysis can be performed using concentrated acid or alkali at high temperature. This process, however, may result in partial degradation or racemisation of some amino acids, including the essential ones (Liardon and Hurrell 1983; Ozols 1990). Hydrolysis in subcritical water or using microwave can be performed in shorter duration and less extreme pH, therefore might hinder these problems (Stenberg et al. 2001; Zhu et al. 2011). Albeit liberating less free amino acids, enzymatic hydrolysis can be performed at lower temperature and neutral or slightly alkaline pH, therefore making operation easier and preventing amino acid racemisation. By modifying hydrolysis conditions, it is also possible to control the degree of hydrolysis and the resulting hydrolysate profile. Protein hydrolysates can be used in food or drink supplements (e.g. sports, weight-control, or geriatrics), or in clinical nutrition (e.g. for patients with allergy or liver disease). As native proteins can sometimes induce allergenic reactions, hydrolysis of the proteins can be used to yield short peptides that are less allergenic and have higher digestibility (Clemente 2000). While proteases have different specificities, it is also possible to selectively hydrolyse specific amino acid bonds or groups of amino acids by selecting different proteases (Tavano 2013).

Another alternative method to modify hydrolysate profiles is using non-aqueous solvents during hydrolysis. Different hydrolysate profiles were observed during casein and β-lactoglobulin hydrolysis in 0–60 % ethanol (Tchorbanov and Iliev 1993; Dalgalarrondo et al. 1995). On the other hand, casein hydrolysis in water-immiscible *n*-hexane, isooctane, and ethyl acetate showed similar hydrolysate profile despite differences in degree of hydrolysis (Sarmento et al. 2006). In non-aqueous solvent, both the substrate and the peptides resulting from cleavage of non-terminal residues have different solubilities compared to solubilities in water. The applied (exo-)protease thus may be exposed to a different part of the protein/peptide, resulting in different free amino acid profiles.

The objective of this research was to selectively produce hydrophobic amino acids from agro-industrial residues. Wheat gluten (as representative of wheat DDGS) and rubber seed protein concentrate were used in the hydrolysis experiments, and the course of hydrolysis was followed in time. Hydrolysis in ethanol was also performed to study the production of free amino acids and the influence of ethanol on selectivity. Experiments with bovine serum albumin (BSA) were used as a reference.

## Materials and methods

### Materials

Rubber seed protein concentrate (48 % protein) was prepared from rubber seed press cake by alkaline extraction of the press cake using 0.1 M NaOH at solid-to-liquid ratio of 1:10 (*w*/*v*), 25 °C, for 1 h, followed by precipitation at pH 5 (4 °C, 24 h) and freeze drying. Wheat gluten was obtained from Cargill (the Netherlands). BSA and Alcalase 2.4L FG were obtained from Sigma-Aldrich (USA). Validase FP concentrate, Pronase, and Peptidase R were obtained from DSM (the Netherlands), Roche Diagnostics (Germany), and Amano (Japan), respectively. Chemicals used were of analytical grade.

### Solubility of rubber seed proteins at different pH

Solubility of rubber seed proteins was determined according to Morr et al. (1985). Rubber seed protein concentrate was dispersed in water to get a homogeneous mixture with final concentration of 1 % (*w*/*w*) protein. The pH was adjusted to the desired pH (1 through 13) using 6 and 0.1 M HCl or NaOH. The mixture was stirred at 250 rpm, 25 °C (2mag magnetic stirrer, Germany) for 1 h, followed by centrifugation at 3000×*g*, 20 °C, for 30 min. The supernatant was separated and analysed for protein content.

The experiment was performed in triplicate. Solubility (%) was calculated as the weight of dissolved protein in the supernatant divided by the total protein weight in the mixture.

### Solubility of rubber seed proteins at different ethanol concentration

Rubber seed protein concentrate was dispersed in water at the concentration of 3 % (*w*/*w*) protein, and the pH was adjusted to pH 8.5 using 6 and 0.1 M NaOH. To this mixture, water and ethanol were subsequently added to get 10–70 % (*w*/*w*) ethanol concentration and final protein concentration of 1 % (*w*/*w*). The mixture was stirred at 250 rpm, 25 °C (2mag magnetic stirrer, Germany) for 1 h, followed by centrifugation at 3000×*g*, 20 °C, for 30 min. The supernatant was separated and analysed for protein content.

The experiment was performed in triplicate. Solubility calculation was similar to solubility at different pH.

### Enzymatic protein hydrolysis using proteases combinations

To study the hydrolysis of our selected substrates, four combinations of protease mixtures were tested (Table 1), based on results of previous experiments with wheat gluten (Sari et al. 2014). Validase FP concentrate and Pronase are mixtures of endo- and exo-proteases with broad specificity. Peptidase R yielded the highest free amino acids compared to other exo-proteases tested. Alcalase 2.4L FG was also selected due to reported specificity towards hydrophobic amino acids (Kasper et al. 2014).

**Table 1:** Hydrolysis conditions

Proteases combination	pH	*t* = 0–1.5 h			*t* = 1.5–24 h		
		Protease	Activity^a^	*T* (°C)	Protease	Activity^a^	*T* (°C)
Validase 2×	7	Validase FP concentrate	400,000 HU/g^b^	55	Validase FP concentrate	400,000 HU/g^b^	55
Validase + Peptidase	7	Validase FP concentrate	400,000 HU/g^b^	55	Peptidase R	420 U/g^d^	40
Pronase + Peptidase	7	Pronase	7000 U/g^c^	55	Peptidase R	420 U/g^d^	40
Alcalase 2×	8.5	Alcalase 2.4L FG	900 U/g^c^	55	Alcalase 2.4L FG	900 U/g^c^	55

^a^The activity as given by the supplier

^b^HU = haemoglobin unit

^c^Unit determined by non-specific protease assay, 1 U will hydrolyse casein to produce colour equivalent to 1.0 μmol of tyrosine per minute

^d^Unit determined by L-Leucyl-Glycyl-Glycine method

Rubber seed protein concentrate was dispersed in water to get a mixture with concentration of 5 % (w-protein/w-solvent). The pH was adjusted to fit the protease optima (Table 1) using 6 and 0.1 M NaOH, and Britton-Robinson buffer was added at 0.01 M. The mixture was stirred at 250 rpm (2mag magnetic stirrer, Germany). The optimal temperature (see Table 1) was kept with a circulating water bath (Julabo). After 30 min, protease at 1 % *w*/w-protein was added and time was set as *t* = 0. Another 1 % protease was added at *t* = 1.5 h to a total protease concentration of 2 %. Samples were taken at *t* = 0, 1, 3, 6, 9, and 24 h. To inactivate the protease after reaction, the sample tubes were incubated at 90 °C for 10 min and stored on ice immediately thereafter, until centrifuged at 7000×*g*, 4 °C for 20 min. The supernatant was separated and filtered through a 0.45-μm Minisart filter to remove insoluble matter. BSA was hydrolysed in a similar procedure using a combination of Pronase and Peptidase R. The experiments were performed in triplicates. Identical experiments without protease addition were performed as control.

Wheat gluten was hydrolysed with all protease combinations in Table 1. The experiments were carried out in duplicates as described previously (Sari et al. 2014); experimental set-ups were similar to experiments with rubber seed protein concentrate except no buffer was added and the experiments with Validase FP concentrate was performed at pH 6.

### Enzymatic protein hydrolysis in ethanol

Rubber seed protein concentrate or BSA was dispersed in water at the concentration of 2.5 % (w-protein/w-solvent), and the pH was adjusted to the desired pH using 6 and 0.1 M NaOH. Water, ethanol, and Pronase dissolved in 0.1 M Britton-Robinson buffer were subsequently added to get the final concentrations of 1 % (*w*/*w*) protein, 0–50 % (*w*/*w*) ethanol, and 5 % w-protease/w-protein. The mixture was incubated at 55 °C for 24 h. To inactivate the protease after the reaction, the sample tubes were incubated at 90 °C for 10 min and stored on ice immediately, until centrifuged at 7000×*g*, 4 °C for 20 min. The supernatant was separated and filtered through the 0.45-μm Minisart filter to remove insoluble matter. The experiment with rubber seed protein concentrate was performed in triplicate and the experiment with BSA was performed in duplicate.

### Analysis

The analysis was performed once for each sample. The analysis was repeated when the standard deviations of replicate treatments were higher than 10 % of the mean value.

#### Protein content

Kjeldahl and modified Lowry methods were applied to measure protein content in determination of rubber seed proteins’ solubility. Kjeldahl results were calculated with nitrogen-to-protein conversion factor of 5.7 (Widyarani et al. 2014).

The modified Lowry method (Peterson 1977) was applied to determine protein content in the hydrolysate, as this method only requires samples in small volume, and therefore enables frequent sampling during the experiment. In the presence of free amino acids, the calculation for protein concentration was modified as discussed in “Results” section.

$$ Protein\  concentration= measured\  soluble\  protein\kern-0.1em + free\  amino\  acids - tyrosine - tryptophan $$

All units are in milligram-protein per millilitre.

#### Degree of hydrolysis

Degree of hydrolysis was determined using a modified OPA method (Nielsen et al. 2001). Based on amino acid composition, the total peptide bonds were 7.8 meqv/g for rubber seed proteins, 7.5 meqv/g for wheat gluten, and 8.1 meqv/g for BSA.

#### Amino acid composition

To measure amino acid composition of the substrates, samples were first acid hydrolysed at 110 °C for 24 h using 6 M HCl containing 1 % (*w*/*v*) phenol (Meussen et al. 2014). Alkaline hydrolysis (4.2 M NaOH, 110 °C, 24 h) was performed specifically for tryptophan determination (Allred and MacDonald 1988). The hydrolysates were dissolved in methanol and filtered through a 0.2-μm Minisart filter; this procedure was also applied to the hydrolysates from the experiments to measure free amino acids. The filtered solutions were loaded onto Ultra-HPLC Dionex RSLC (Dionex Corporation, USA) where the amino acids were separated using an Acquity UPLC BEH C18 reversed phase column. Norleucine was used as standard. Detection was performed at 263 and 338 nm (Meussen et al. 2014).

### Statistical analysis

The values of different treatments were compared using Student’s *t* test or ANOVA with LSD post hoc analysis; *p* < 0.05 was regarded as significant.

## Results

### Amino acid composition

The three substrates used in our experiments contained comparable amounts of hydrophobic amino acids. Valine, proline, and leucine were the hydrophobic amino acids with the highest fraction in rubber seed protein concentrate, wheat gluten, and BSA, respectively (Table 2). Hydrophobic amino acids are predominantly present in the interior of the protein (Tanford 1962), as this conformation stabilises the protein in aqueous solution. To enable contact between hydrophobic amino acids and the protease, the protein must be unfolded.

**Table 2:** Amino acid side chain hydrophobicity (Δ*f*) and amino acid composition of rubber seed protein concentrate, BSA, and wheat gluten

Amino acid^a^ (AA)	Abbreviation	Δ*f* ^b^ (cal/mol)	Amino acid fraction (mol/mol-total amino acids)		
			Rubber seed protein concentrate	Wheat gluten	BSA
Phenylalanine	Phe	2650	0.04	0.03	0.05
Leucine	Leu	2420	0.08	0.07	0.12
Isoleucine	Ile	2970	0.04	0.04	0.02
Tyrosine	Tyr	2870	0.02	0.02	0.04
Tryptophan	Trp	3220	0.01	0.01^c^	0.00
Valine	Val	1690	0.11	0.04	0.07
Methionine	Met	1300	0.01	0.02	0.01
Proline	Pro	2600	0.06	0.15	0.05
Cystine/cysteine	Cys	1000^d^	0.00	0.00	0.00
Alanine	Ala	500	0.08	0.04	0.09
Glycine	Gly	0	0.08	0.06	0.03
Threonine	Thr	400	0.04	0.03	0.06
Serine	Ser	−300	0.07	0.06	0.05
Lysine	Lys	1500^e^	0.02	0.04	0.10
Histidine	His	450	0.02	0.01	0.03
Glutamic acid/glutamine	Glx	550^f^	0.13	0.33	0.14
Aspartic acid/asparagine	Asx	540^g^	0.12	0.03	0.10
Arginine	Arg	730	0.09	0.02	0.04
Total hydrophobic amino acids^h^			0.35	0.37	0.35

^a^The amino acids are listed from the most hydrophobic (phenylalanine) to the least hydrophobic (arginine) as calculated with phase-partitioning constants of molecular fragments (Black and Mould 1991)

^b^Δ*f* (hydrophobicity) = free energy change for transfer from ethanol to water at 25 °C (Tanford 1962; Nozaki and Tanford 1971). Values for ethanol were selected instead of average values of organic solvents due to the relevance with our experiment

^c^Calculated from Woychik et al. (1961)

^d^Data from Bigelow (1967)

^e^The high hydrophobicity of lysine is due to the presence of norleucine side chain that is very hydrophobic (∆*f* = 2700 cal/mol). However, as lysine is positively charged, it is not grouped as hydrophobic

^f^Value for glutamic acid

^g^Value for aspartic acid

^h^Phenylalanine, leucine, isoleucine, tyrosine, tryptophan, valine, methionine, proline (Black and Mould 1991)

### Protein solubility

**Fig. 1:**
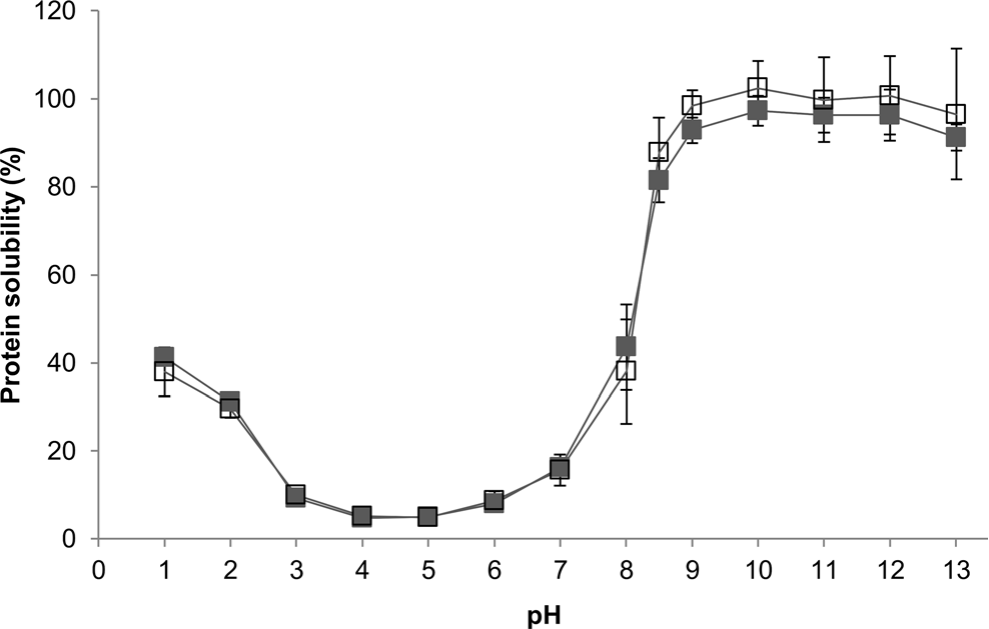
Solubility of rubber seed protein at 25 °C as a function of pH, as determined by modified Lowry (*unfilled square*) and Kjeldahl (*filled square*) methods

Solubility of rubber seed proteins at different pHs was measured to indicate the available protein fraction in the solution at the start of hydrolysis. At pH 7, where some of the experiments were conducted (Table 1), only 16 % of protein was soluble. Protein concentrate was prepared using alkaline extraction; therefore, it consisted mostly of alkaline-soluble fractions. As expected, most of the proteins were soluble at pHs up and above 8.5 (Fig. 1). The lowest solubility in water occurred between pH 4 and 5, which indicates its isoelectric point. BSA is fairly soluble at pH 7 (Elysée-Collen and Lencki 1997), with isoelectric point at pH 5 (Conway-Jacobs and Lewin 1971). Wheat gluten solubility is less than 5 % at pH 7, which is estimated as its isoelectric point (Wang et al. 2006).

**Fig. 2:**
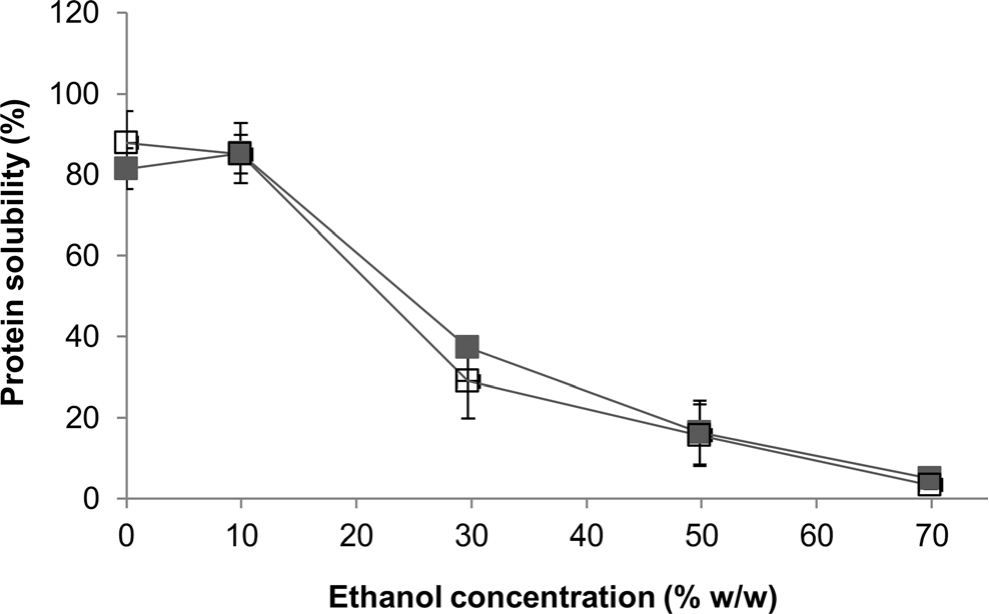
Solubility at different ethanol concentrations for rubber seed proteins at pH 8.5, 25 °C, as determined by modified Lowry (*unfilled square*) and Kjeldahl (*filled square*)

Protein denaturation reduces protein solubility; however, the conformational change may expose the interior amino acids to the proteases. There was no significant difference (*p* > 0.05) of rubber seed proteins solubility between 0 and 10 % *w*/*w* ethanol (Fig. 2), but solubility decreased at higher ethanol concentrations, indicating the protein was denatured. BSA was completely soluble in water up to 0.56 g-BSA/g-solution, and the solubility did not change in up to 30 % *w*/*w* ethanol. At 37 % *w*/*w* ethanol, complete solubility of 0.05 g-BSA/g-solution was still observed (Elysée-Collen and Lencki 1997). The use of 50–65 % *v*/*v* ethanol is reported to even increase wheat gluten solubility from 2 to 37 g-gluten/l-solvent (Robertson et al. 1999).

Due to the low detection limit (up to 0.4 mg/ml), protein determinations by modified Lowry had higher standard deviations at high protein concentrations. However, comparison between this method and Kjeldahl for rubber seed proteins shows good correlation based on linear regression (*R*^2^ = 0.986):

$$ \mathrm{Kjeldahl}\;\mathrm{solubility}\;\left(\%\right)=0.93\;\mathrm{Lowry}\;\mathrm{solubility}\;\left(\%\right)+2.43\% $$

Free amino acids other than tyrosine and tryptophan may not be detected with Lowry (Peterson 1979). However, this method requires only small sample volume that enables frequent sampling during the experiment. Based on these results, we used the modified Lowry method (Peterson 1977) corrected with free amino acids concentrations from HPLC measurements to determine protein contents of the hydrolysates.

### Hydrolysis with protease combinations

#### Influence of protease

**Fig. 3:**
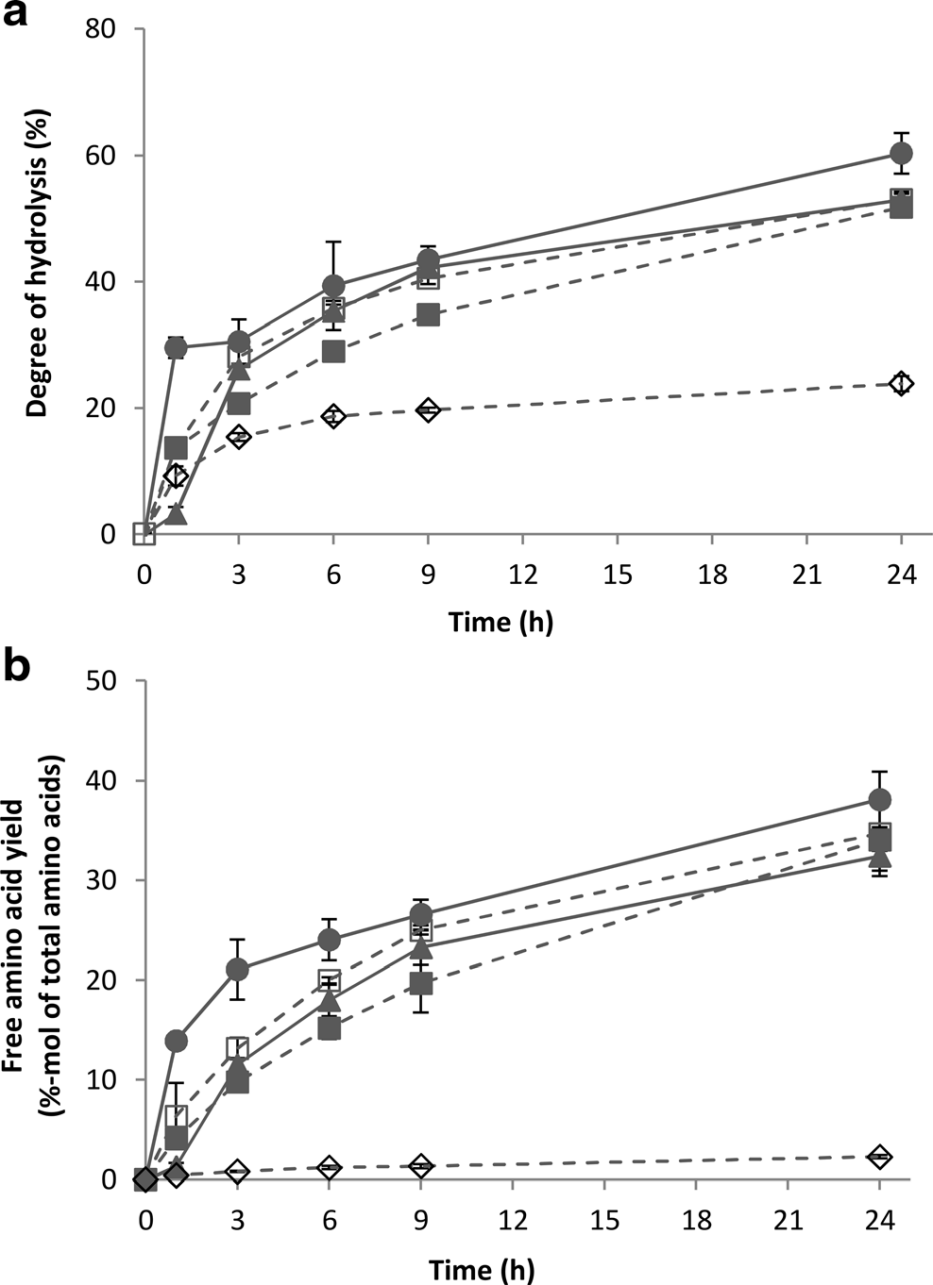
Degree of hydrolysis (**a**) and free amino acid yield (**b**) during 24 h hydrolysis of BSA with Pronase + Peptidase (*filled circle*) and hydrolysis of rubber seed proteins with Validase 2× (*unfilled square*), Validase + Peptidase (*filled square*), Pronase + Peptidase (*filled triangle*), and Alcalase 2× (*unfilled diamond*)

**Fig. 4:**
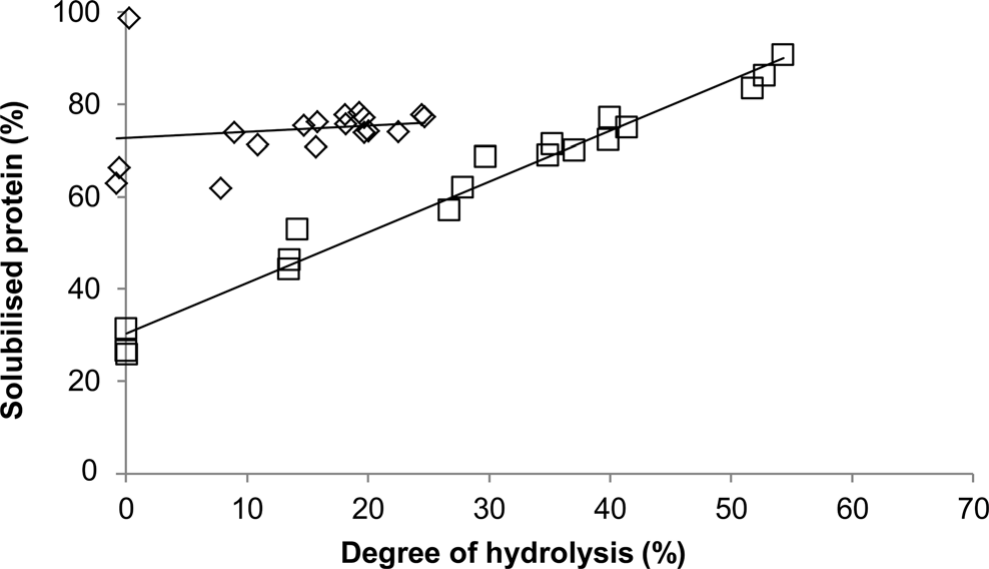
Protein solubility as a function of degree of hydrolysis during 24 h hydrolysis of rubber seed proteins with Validase 2× (*unfilled square*) and Alcalase 2× (*unfilled diamond*). The lines have a different starting point because of the different pH’s of the mixtures (7 versus 8.5)

Figure 3a, b shows, respectively, the degree of hydrolysis and the yield of liberated free amino acids relative to the total available amino acids in the experiment. After 24 h hydrolysis of rubber seed proteins, comparable degree of hydrolysis and free amino acid yield were observed for the three protease combinations at pH 7: Validase 2×, Validase + Peptidase, and Pronase + Peptidase. With increasing degree of hydrolysis, the amount of solubilised protein for these experiments also increased (Fig. 4). We previously observed this in experiments with wheat gluten (Sari et al. 2014). Up to *t* = 3 h, the increase in protein solubility was mainly attributed to the formation of peptides. Material balance between fractions and the high degree of hydrolysis in all experiments suggests that the peptides were very short-chained and probably mainly present as di- or tri-peptides. After 3 h, the increase in protein solubility was the result of free amino acids liberation.

Despite the higher solubility of rubber seed proteins at pH 8.5 (Fig. 1), the experiment with Alcalase 2× gave the lowest degree of hydrolysis (Fig. 3a). Alcalase 2.4L FG is an endo-protease from *Bacillus licheniformis* that has lower activity compared to the other proteases (Table 1); therefore, the amount of liberated free amino acids was lower than the other experiments (Fig. 3b). Furthermore, low exo-protease activity and inhibition of proteases from *B. licheniformis* by short peptides have been reported (Kasper et al. 2014). This is consistent with our results that the hydrolysate entailed mostly peptides and less free amino acids. As free amino acids were partially accountable for the increase in protein solubility, the amount of solubilised protein for the Alcalase 2× experiment also did not change even though the degree of hydrolysis increased during the 24 h (Fig. 4).

#### Influence of substrate composition

**Fig. 5:**
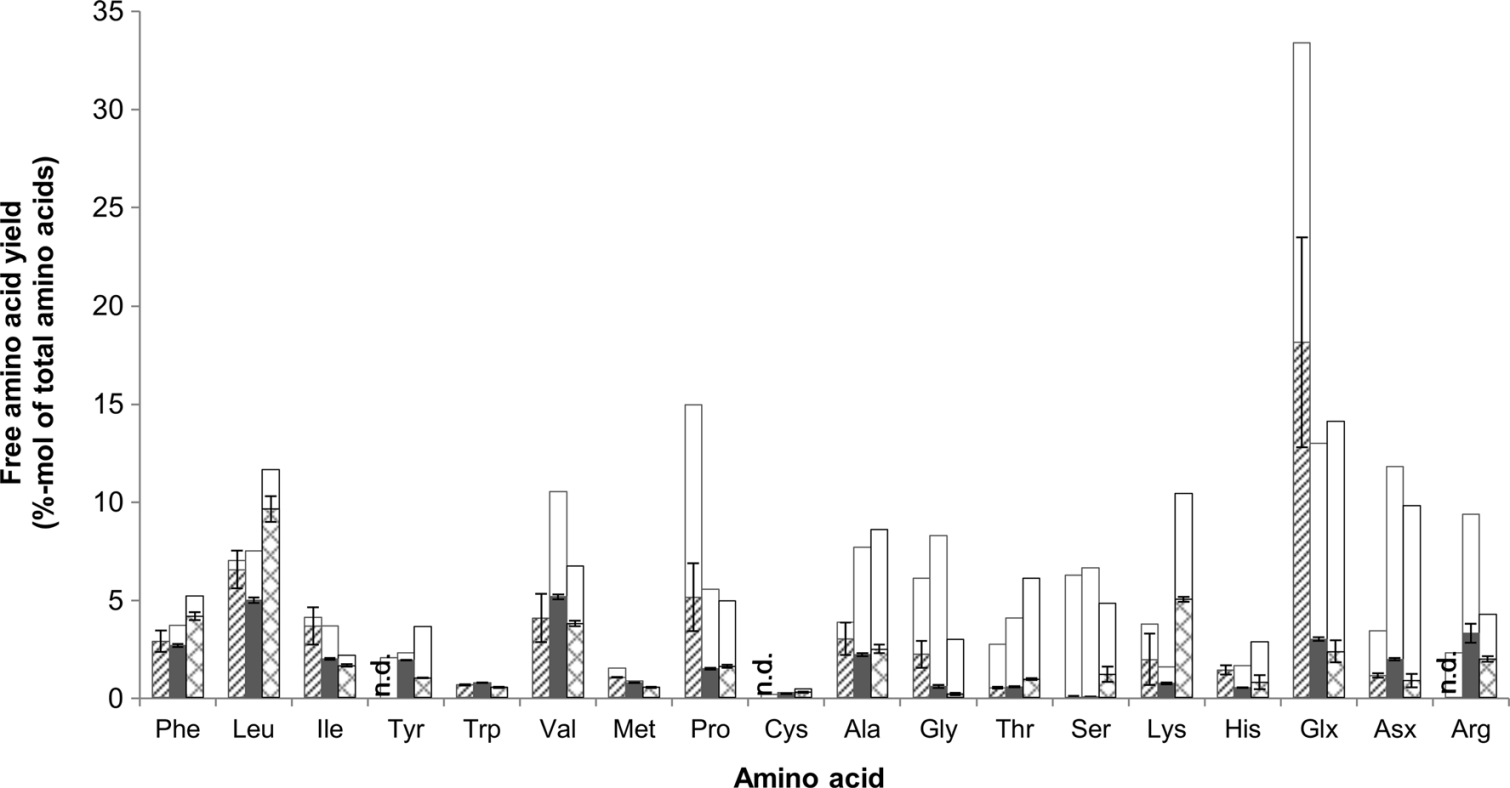
Free amino acid yield after 24 h hydrolysis of wheat gluten (*square with upward diagonal lines*), rubber seed proteins (*filled square*), and BSA (*square outlined with diamond*) with Pronase + Peptidase; *unfilled bars* indicate the available amino acid in the substrate. *n*.*d*. value below detection level. The values expressed in this figure can be found in Table S1 in the Supplementary Material

After 24 h hydrolysis with Pronase + Peptidase, the free amino acid yield from wheat gluten was 52 ± 13 % of total amino acids, which was higher than both rubber seed proteins (32 ± 2 %) and BSA (38 ± 3 %). Figure 5 shows the yield of individual amino acids based on the total amino acids available in the substrates. For all amino acids except lysine and proline, different yields between substrates were observed (Fig. 5; Table S1), indicating that substrate composition influenced the liberation of amino acids during hydrolysis.

Previous studies have shown that combination of endo- and exo-proteases leads to higher degree of hydrolysis and yields more free amino acids (Kamnerdpetch et al. 2007; Sari et al. 2014). This was further illustrated when in our experiments the amount of free amino acids from wheat gluten in the experiment with Validase + Peptidase combination was higher than in the Validase 2× combination (Sari et al. 2014). For rubber seed proteins, on the other hand, the amount of free amino acids was similar or even higher (*t* = 3 and 6 h) for the Validase 2× combination than the Validase + Peptidase combination (Fig. 3b). Peptidase R has a high proline-specific aminopeptidase activity (Kilcawley et al. 2002), which suggests that the difference might be attributed to the amount of proline in wheat gluten (0.15 mol/mol-total amino acid) that was almost three times higher than that in rubber seed proteins (Table 2, Fig. 5). This might also explain the higher free amino acid yield of wheat gluten compared with BSA, as the latter also has low proline content.

#### Hydrophobic amino acids yield

**Fig. 6:**
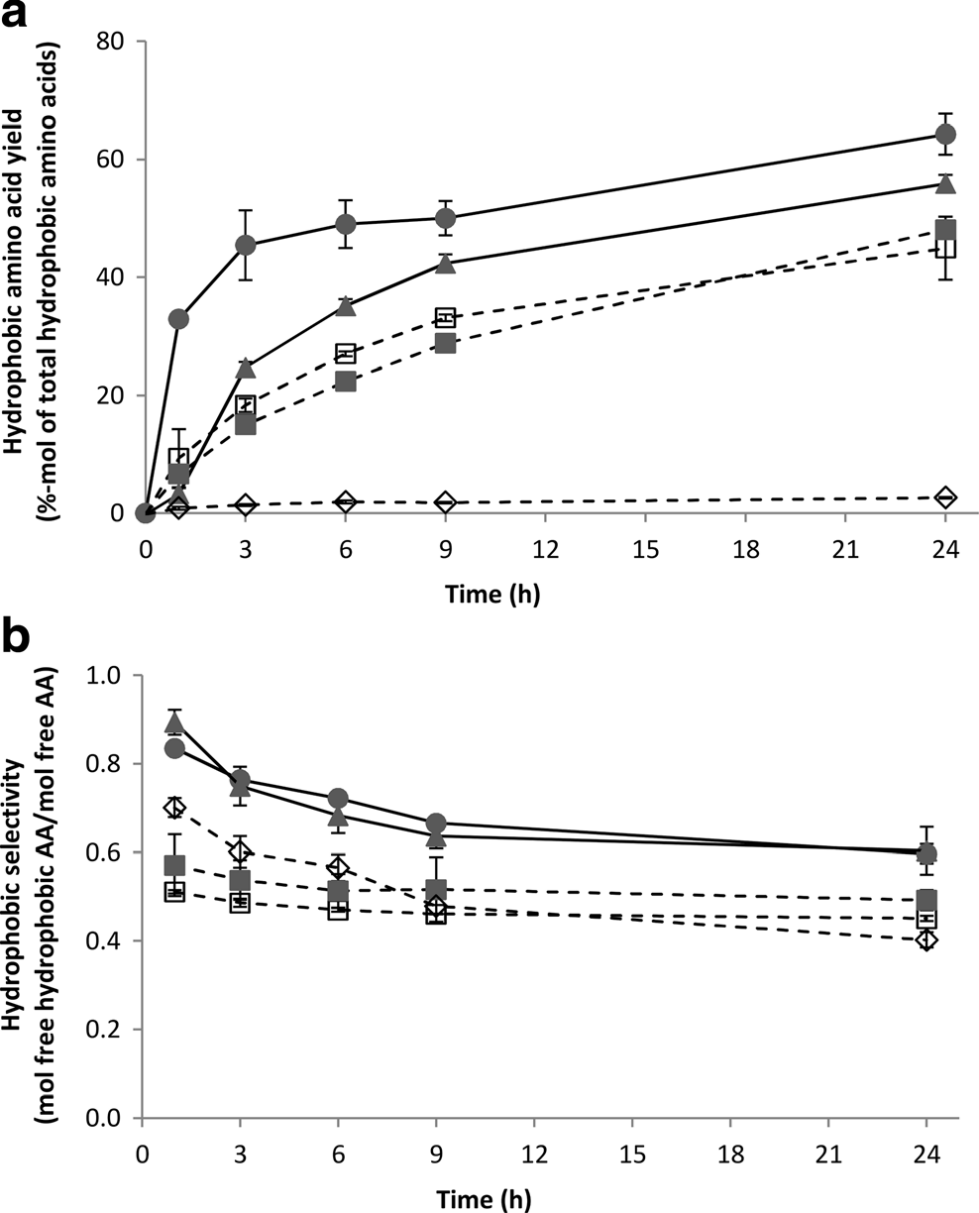
Hydrophobic amino acid yield (**a**) and selectivity (**b**) during 24 h hydrolysis of BSA with Pronase + Peptidase (*filled circle*) and hydrolysis of rubber seed proteins with Validase 2× (*unfilled square*), Validase + Peptidase (*filled square*), Pronase + Peptidase (*filled triangle*), and Alcalase 2× (*unfilled diamond*)

Figure 5 shows that not all amino acids were liberated to the same degree. During hydrolysis of rubber seed protein concentrate, each protease combination resulted in different hydrophobic amino acid yield and selectivity. After 24 h of hydrolysis, 45–56 % of the total hydrophobic amino acids in the substrate could be recovered in the hydrolysate (Fig. 6a), higher than the overall free amino acid yield compared to the total amino acids (Fig. 3b).

Hydrophobic selectivity is defined as the amount of free hydrophobic amino acids: phenylalanine, leucine, isoleucine, tyrosine, tryptophan, valine, methionine, and proline (Black and Mould 1991), relative to the total liberated free amino acids on molar-base. Selectivity for each combination was highest at *t* = 1 h and decreased over time, except for the Validase + Peptidase combination (Fig. 6b). There was no significant difference (*p* > 0.05) of hydrophobic selectivity between Validase FP concentrate with and without Peptidase R, except for *t* = 24 h (Fig. 6b). Furthermore, the higher selectivity of Pronase compared to Validase FP concentrate was already observed at *t* = 1 h when only Validase FP concentrate or Pronase was added and no second protease mixture. Pronase is a non-specific protease mixture. The hydrophobic selectivity might be attributed to the presence of leucine aminopeptidase (Narahashi 1970). This is consistent with our results showing that free leucine, phenylalanine, and valine were the amino acids that contributed most to the selectivity.

Alcalase 2.4L FG is considered to have specificity towards hydrophobic amino acids (Kasper et al. 2014), and its selectivity increases as the peptide size decreases (Gallegos-Tintoré et al. 2011). In our experiments, however, the selectivity of Alcalase 2.4L FG after 24 h was lower than the other protease combinations (Fig. 6b). This might be because even though Alcalase cleaved peptide bonds next to hydrophobic amino acids, it did not always liberate free amino acids due to the lack of exo-protease activities.

For the Pronase + Peptidase combination, comparison between the hydrophobic amino acid fraction in the substrate and selectivity in the hydrolysate at *t* = 6 h (Table 3) shows a twofold increase of selectivity for rubber seed proteins and BSA hydrolysates. For wheat gluten, a slightly less 1.5 times increase was observed. For the same protease combination, at *t* = 24 h, hydrophobic selectivity for rubber seed proteins and BSA hydrolysates were both still high at 0.60 mol/mol, while wheat gluten hydrolysate was only 0.46 mol/mol. The difference might be attributed to the high amount of liberated glutamic acid/glutamine from wheat gluten. The glutamic acid/glutamine fraction in wheat gluten was 0.33 mol/mol-total amino acid (Table 2) and the liberated glutamic acid/glutamine at *t* = 6 h and *t* = 24 h were 0.20 and 0.35 mol/mol-total free amino acid, respectively, which significantly dominated the hydrolysate profile. Similar influence of glutamic acid/glutamine on wheat gluten hydrolysis was also observed for the Validase + Peptidase combination (Table 3; Sari et al. 2014).

**Table 3:** Hydrophobic amino acid selectivity (mol free hydrophobic amino acid/mol-total free amino acid)

Substrate	Hydrophobic amino acid fraction in the substrate	Protease					
		Validase + Peptidase		Pronase + Peptidase		Pronase	Pronase, 10 % ethanol
		6 h	24 h	6 h	24 h	24 h	24 h
Rubber seed proteins	0.35	0.51 ± 0.04	0.49 ± 0.02	0.68 ± 0.04	0.60 ± 0.05	0.55 ± 0.05	0.56 ± 0.06
Wheat gluten	0.37	0.42^a^	0.47 ± 0.03	0.56^a^	0.46 ± 0.02	n.a.	n.a.
BSA	0.35	n.a.	n.a.	0.72 ± 0.00	0.60 ± 0.02	0.40 ± 0.00	0.45 ± 0.00

*n*.*a*. data not available

^a^Value from one measurement

### Hydrolysis in ethanol

**Fig. 7:**
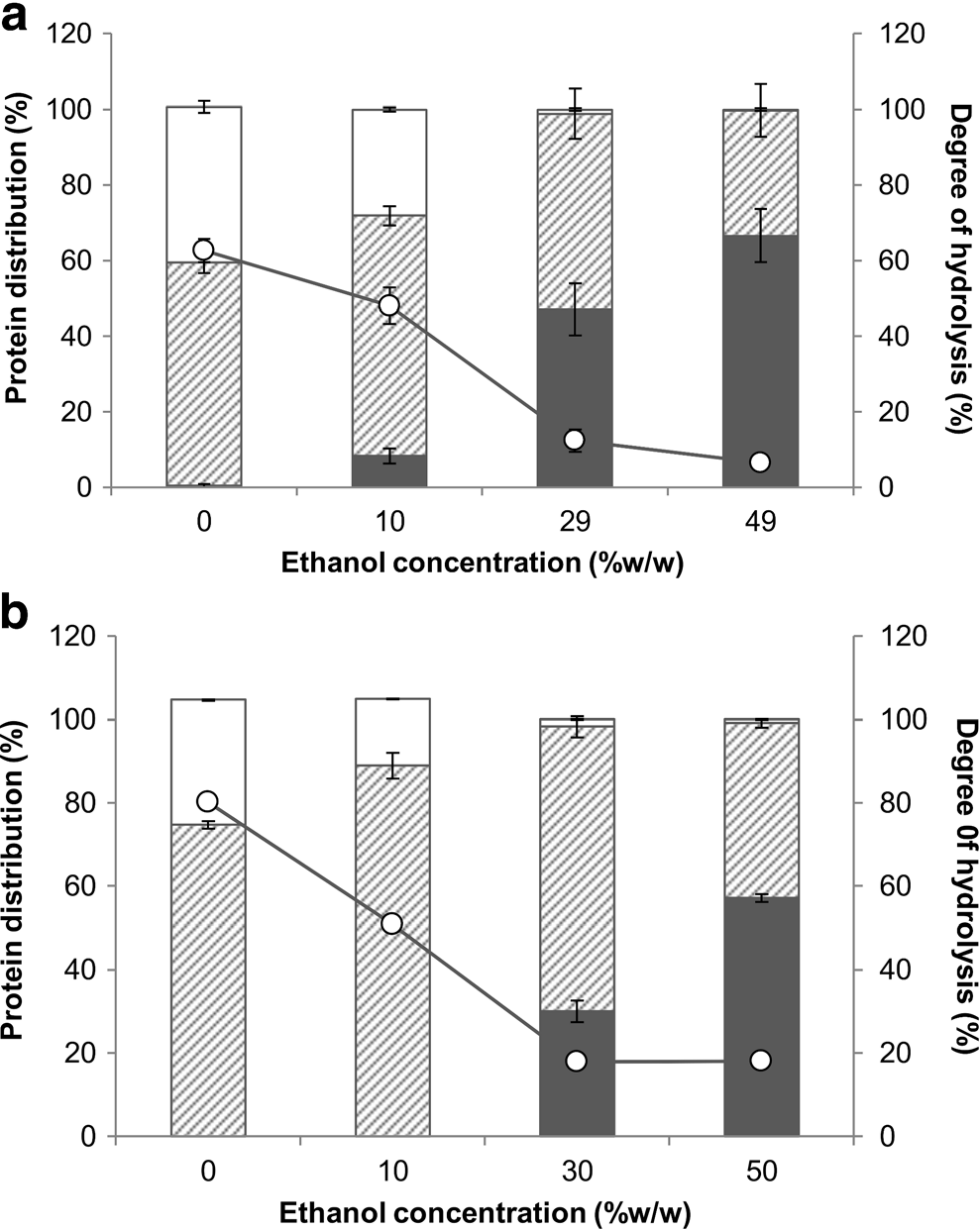
Degree of hydrolysis (*unfilled circle*) and protein molar distribution between insoluble (*filled square*), peptide (*square with upward diagonal lines*), and free amino acid (*unfilled square*) fractions after 24 h hydrolysis of rubber seed proteins (**a**) and BSA (**b**) using Pronase at different ethanol concentrations

Hydrolysis in ethanol was performed to establish ethanol influence on amino acids yield and selectivity. Figure 7a, b shows that at 10 % ethanol, around 50 % degree of hydrolysis could still be obtained. As much as 28 and 16 % of the original protein from rubber seed proteins and BSA, respectively, were liberated to free amino acids. This shows that the Pronase was still active at 10 % ethanol, albeit at lower activity. At 30 % ethanol, however, not only did the free amino acid yield decrease compared to the experiments at 0 and 10 % ethanol but also the protein solubility was similar (for rubber seed proteins) or lower (for BSA) than in the experiments without protease. Here, the protease itself can be denatured and may have formed an insoluble complex with the peptides (Widyarani et al. 2014).

**Fig. 8:**
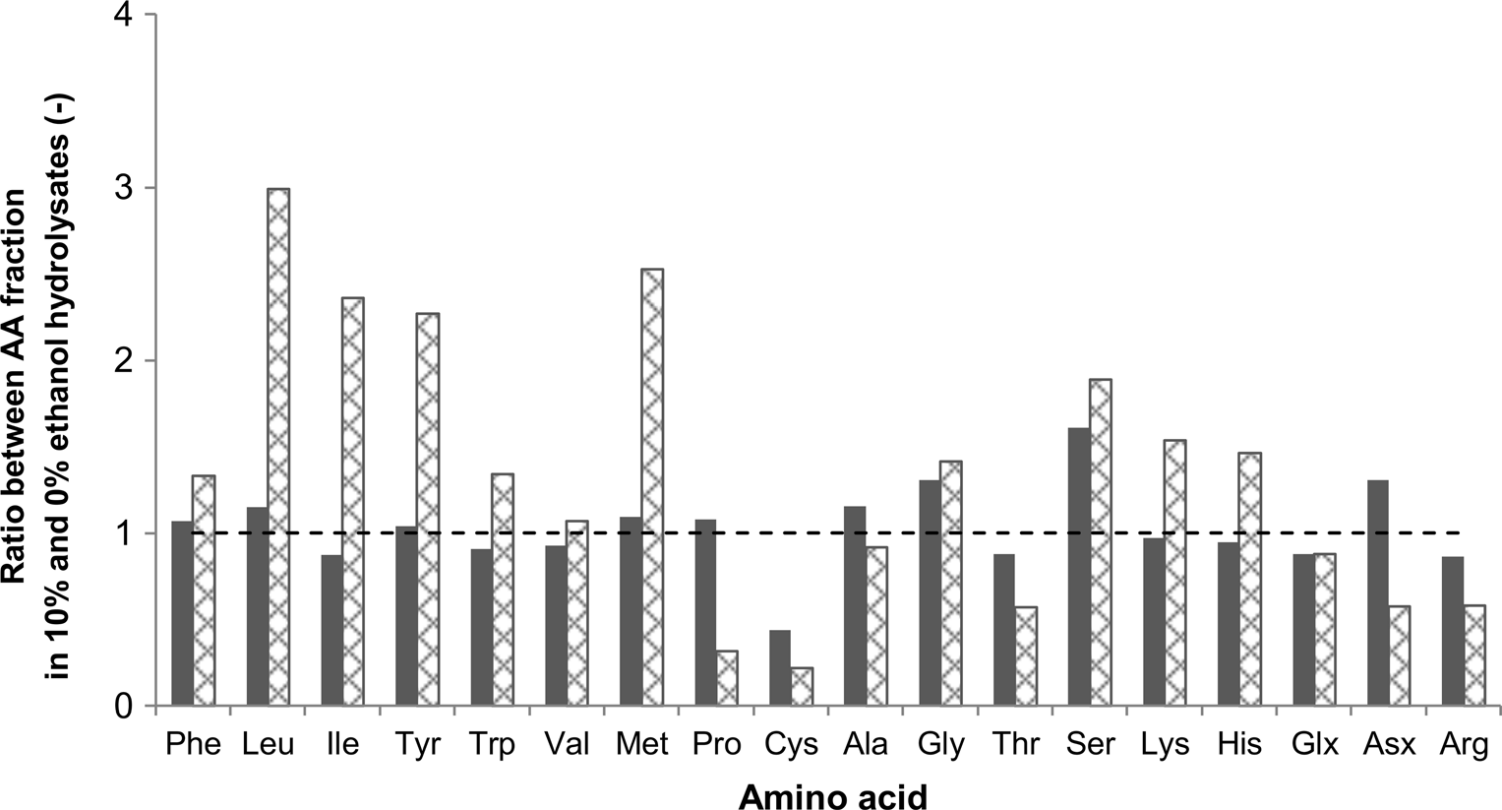
Ratio of free amino acid fraction in 10 % ethanol hydrolysate to the one in 0 % ethanol hydrolysate after 24 h hydrolysis of rubber seed proteins (*filled square*) and BSA (*square outlined with diamond*) with Pronase. The *dashed line* indicates a ratio of 1, when the amino acid fraction in 10 % ethanol hydrolysate was equal to the one in the 0 % ethanol hydrolysate

It was expected that at higher ethanol concentrations, hydrophobic selectivity could be higher, even when the total free amino acid yield was lower. The selectivity increase, however, was only observed for BSA between 0 and 10 % ethanol (Table 3). Comparison between free amino acid fractions in 10 % ethanol hydrolysate and 0 % ethanol hydrolysate (Fig. 8) shows no clear pattern of ethanol influence on free amino acid composition in the hydrolysate. Protein conformational change due to ethanol may expose other parts in different proteins and in a different fashion compared to when ethanol was not present. Also, the protease we used was a mixture of several enzymes that each may respond differently to ethanol presence. At higher ethanol concentrations both selectivity and yield decreased, which shows that ethanol addition could not be used to increase selectivity for protein hydrolysis into free hydrophobic amino acids.

Degree of hydrolysis of BSA at 0 % ethanol was 80 % (Fig. 7b), suggesting that most of the proteins were completely hydrolysed. However, only 30 % protein was liberated to free amino acids. This either suggests that the amount of free amino acids was underestimated or some secondary hydrolysate products were formed, e.g. pyroglutamic acid or diketopiperazine (Hirs et al. 1960; Baxter et al. 2004). In the presence of both leucine aminopeptidase and carboxypeptidase, terminal proline can form diketopiperazine instead of being liberated as free proline (Hirs et al. 1960; Smyth and Elliott 1964). Indeed, we observed that the amount of free proline in the hydrolysate was very low. The presence of protease with proline-aminopeptidase activity, e.g. Peptidase R, may surmount diketopiperazine formation. This is consistent with the results for experiments with a combination of Pronase + Peptidase.

## Discussion

There are three points where hydrophobic selectivity can be achieved, namely selection of starting material with high hydrophobic amino acids, selection of hydrolysis conditions, and separation of the final hydrolysate. The amounts of hydrophobic amino acids for the three substrates used in our experiments were 0.35, 0.37, and 0.35 mol/mol-total amino acid for rubber seed protein concentrate, wheat gluten, and BSA, respectively. These values are higher than, e.g. soybean, sunflower, and *Jatropha* seed press cake/meal and protein isolate, which have 0.30–0.33 mol-hydrophobic/mol-total amino acid, but close to rapeseed meal with 0.34 mol-hydrophobic/mol-total amino acid (Tranchino et al. 1983; Conde et al. 2005; Devappa and Swamylingappa 2008; Frikha et al. 2012; Fleddermann et al. 2013). On the other hand, the hydrophobic fraction of wheat gluten is still lower than corn gluten meal that has 0.43 mol-hydrophobic/mol-total amino acid (Jin et al. 2015).

Our results show that the amount of free hydrophobic amino acids in hydrolysate relative to the total free amino acids was influenced by the extent of hydrolysis and protease selection. Prolonged incubation increased the overall free amino acid yield, but decreased the selectivity towards hydrophobic amino acids. In our experiments, the highest selectivity towards hydrophobic amino acids was obtained by combining Pronase and Peptidase R; selectivity of 0.6 mol/mol-total free amino acid was observed after 6 h hydrolysis of wheat gluten and 24 h hydrolysis of rubber seed proteins and BSA. Pronase has both endo- and exo-protease activity, and it also showed high hydrophobic selectivity without the presence of Peptidase R, an exo-protease. On the other hand, hydrolysis of potato pulp using combinations of Alcalase or Novo Pro-D as endo-protease and Flavourzyme or Corolase LAP as exo-protease showed higher hydrophobic selectivity of Corolase, regardless of the endo-protease (Kamnerdpetch et al. 2007). Experiments with Pronase without Peptidase R addition also showed the possibility of secondary products formation. Therefore, in order to achieve high hydrophobic selectivity, selection of the appropriate exo-protease is crucial. Based on our results and on potato pulp hydrolysis results from literature (Kamnerdpetch et al. 2007), we conclude that combination of Pronase and Corolase LAP may yield hydrolysates with high hydrophobic selectivity.

Both rubber seed proteins and BSA were still soluble at 10 % ethanol; this property was hypothesised to be important during hydrolysis. Indeed, around 50 % degree of hydrolysis could still be obtained. On the other hand, results of β-casein and β-lactoglobulin hydrolysis suggest that protein structure is more important as proteins with different structures follow different denaturation patterns (Dalgalarrondo et al. 1995). Both β-casein and β-lactoglobulin are fairly soluble in 0–30 % (*v*/*v*) ethanol. However, while β-casein was readily hydrolysed by pepsin at 0–10 % ethanol and less hydrolysis was observed at 20 % ethanol or higher, β-lactoglobulin hydrolysis by pepsin only occurred at ethanol concentration of 20 % or higher. Pepsin has specificity towards aromatic and hydrophobic amino acids, and it was proposed that these amino acids were located in the interior of β-lactoglobulin and were only exposed to pepsin in the presence of ethanol. In contrast, β-casein has an unordered structure and potential cleavage sites were already exposed without denaturation. To optimise hydrolysis and increase selectivity, investigation of the denaturation pattern in the presence of protease, ethanol, and/or other denaturing agents can be of importance.

The use of ethanol did not influence hydrophobic selectivity, except for BSA at 10 % ethanol. A decrease of Pronase activity was observed at 10 % ethanol and higher. Still, based on the degree of hydrolysis, we saw that peptides were formed. Their profile might be influenced by ethanol addition; however, we did not identify the peptides and therefore no conclusion can be drawn. Higher hydrophobic selectivity might be achieved by using proteases that can maintain their activity in the presence of ethanol. Trypsin, α-chymotrypsin, subtilisin DY (Tchorbanov and Iliev 1993), and papain (Saito et al. 1988) still exhibit some hydrolytic activity in the ethanol concentration up to 70 %. Our own preliminary experiments with papain (data not shown), however, showed that the degree of hydrolysis decreased with increasing ethanol concentration and the free amino acid yield was much lower than the yields achieved from proteases used in this experiment.

The use of protease for hydrolysis enables mild processing, thereby avoiding formation of unwanted compounds or even racemisation of amino acids, making the hydrolysates more suitable for food or feed application compared to chemical hydrolysates. We have shown that 50 % degree of hydrolysis from our substrates could be obtained within 24 h, indicating the hydrolysate comprised of short-chained peptides and free amino acids. Hydrolysate with high fraction of hydrophobic amino acids may taste bitter; valine, leucine, isoleucine, phenylalanine, and tyrosine are some amino acids that are considered have bitter taste (Ney 1971). However, bitterness is also influenced by peptide length; free amino acids and di- and tri-peptides are less bitter than peptides with longer chain (Fujimaki et al. 1970; Matoba and Hata 1972). The final hydrolysate profile can be modified by adjusting hydrolysis time.

Rubber seed protein concentrate and wheat gluten had protein contents of 48 and 74 %, respectively. As representative of agro-industrial residues, the results from these substrates were comparable to BSA that was used in its purified form. This illustrates that protease can be applied for hydrolysis of proteins from (impure) agro-industrial residues to obtain free amino acids. Within a biorefinery framework, the next step after hydrolysis by protease would be the separation of the peptides and free amino acids from the hydrolysate mixture. The peptides and essential amino acids can be used for food or feed applications, while the non-essential amino acids can be used for bulk chemicals production. At this separation stage, hydrophobic selectivity can also be achieved, and this will be the topic for a follow-up article.

## Electronic supplementary material

ESM 1 This article contains an electronic supplementary material. (PDF 166 kb)
