# Supplementary material for: Production of hydrophobic amino acids from biobased resources: wheat gluten and rubber seed proteins
Source: Appl Microbiol Biotechnol. 2016 Apr 27;100:7909–20. doi: 10.1007/s00253-016-7441-8 (PMC4989023; doi:10.1007/s00253-016-7441-8)
Supplement: Supplementary file 1 — This article contains an electronic supplementary material. (PDF 166 kb) [file 253_2016_7441_MOESM1_ESM.pdf]

Applied Microbiology and Biotechnology

Supplementary Material

Production of hydrophobic amino acids from biobased resources: wheat gluten and rubber seed proteins

Widyarani[1,2*], Yessie W. Sari[1,3], Enny Ratnaningsih[4], Johan P.M. Sanders[1,5], Marieke E. Bruins[1,5]

[1] Biobased Chemistry and Technology, Wageningen University

[2] Research Centre for Chemistry, Indonesian Institute of Sciences (LIPI)

[3] Biophysics Division, Department of Physics, Bogor Agricultural University

[4] Study Programme of Chemistry, Faculty of Mathematics and Natural Sciences, Institut Teknologi Bandung

[5] Food & Biobased Research, Wageningen UR

[*]Corresponding author:

Tel./fax: +62 21 7560929/+62 21 7560549
E-mail address: widyarani@lipi.go.id (Widyarani).

Table S1 Free amino acid yield, composition, and relative yield after 24 h hydrolysis of rubber seed protein (RSP), wheat gluten (WG), and BSA with Pronase + Peptidase

**Amino acid**[a] **(AA)**	**Substrate composition** (%-mol of total amino acids in the substrate)			**Free amino acid yield** (%-mol of total amino acids in the substrate)			**Hydrolysate composition** (%-mol of total free amino acids in the hydrolysate)			**Relative Yield** (%-mol of the respective amino acid in the substrate)		
	**RSP**	**WG**	**BSA**	**RSP**	**WG**	**BSA**	**RSP**	**WG**	**BSA**	**RSP**	**WG**	**BSA**
Phenylalanine	4	3	5	3	3	4	8	6	11	71	107	79
Leucine	8	7	12	5	7	10	15	13	25	66	94	82
Isoleucine	4	4	2	2	4	2	6	7	4	52	88	73
Tyrosine	2	2	4	2	0	1	6	0	3	81	0	27
Tryptophan	1	1	0	1	1	1	2	1	1	109	415	175
Valine	11	4	7	5	4	4	16	8	10	49	114	56
Methionine	1	2	1	1	1	1	2	2	1	82	62	94
Proline	6	15	5	1	5	2	4	10	4	27	34	32
Cystine/cysteine	0	0	0	0	0	0	1	0	1	63	0	53
Alanine	8	4	9	2	3	2	7	6	6	28	76	29
Glycine	8	6	3	1	2	0	2	4	0	7	36	6
Threonine	4	3	6	1	0	1	2	1	2	13	18	15
Serine	7	6	5	0	0	1	0	0	3	1	0	24
Lysine	2	4	10	1	2	5	2	4	13	43	51	48
Histidine	2	1	3	0	1	1	2	3	2	29	128	27
Glutamic acid/glutamine	13	33	14	3	18	2	9	35	6	23	55	17
Aspartic acid/asparagine	12	3	10	2	1	1	6	2	2	16	33	9
Arginine	9	2	4	3	0	2	10	0	5	35	0	45
